# Multi‐Zone Visco‐Node‐Pore Sensing: A Microfluidic Platform for Multi‐Frequency Viscoelastic Phenotyping of Single Cells

**DOI:** 10.1002/advs.202406013

**Published:** 2024-09-23

**Authors:** Andre Lai, Stefan Hinz, Alan Dong, Michael Lustig, Mark A. LaBarge, Lydia L. Sohn

**Affiliations:** ^1^ UC Berkeley–UC San Francisco Graduate Program in Bioengineering University of California Berkeley CA 94720 USA; ^2^ Department of Population Sciences Beckman Research Institute City of Hope Duarte 91010 USA; ^3^ Department of Electrical Engineering and Computer Sciences University of California Berkeley 94720 USA; ^4^ Department of Mechanical Engineering University of California Berkeley 94720 USA

**Keywords:** mechanophenotyping, microfluidics, single‐cell biomechanics, viscoelasticity

## Abstract

This study introduces multi‐zone visco‐Node‐Pore Sensing (mz‐visco‐NPS), an electronic‐based microfluidic platform for single‐cell viscoelastic phenotyping. mz‐visco‐NPS implements a series of sinusoidal‐shaped contraction zones that periodically deform a cell at specific strain frequencies, leading to changes in resistance across the zones that correspond to the cell's frequency‐dependent elastic G′ and viscous G″ moduli. mz‐visco‐NPS is validated by measuring the viscoelastic changes of MCF‐7 cells when their cytoskeleton is disrupted. mz‐visco‐NPS is also employed to measure the viscoelastic properties of human mammary epithelial cells across the entire continuum of epithelial transformation states, from average‐ and high‐risk primary epithelial cells, to immortal non‐malignant (MCF‐10A), malignant (MCF‐7), and metastatic (MDA‐MB‐231) cell lines. With a throughput of 600 cells per hour and demonstrated ease‐of‐use, mz‐visco‐NPS reveals a remarkable level of single‐cell heterogeneity that would otherwise be masked by ensemble averaging.

## Graphical abstract

Multi‐zone node‐pore sensing (mz‐visco‐NPS) is an innovative method to measure single‐cell viscoelasticity. This study demonstrates that mz‐visco‐NPS can measure viscoelastic changes of cells and viscoelastic properties of human mammary epithelial cells across a continuum of epithelial transformation states. With a throughput of 600 cells per hour and demonstrated ease‐of‐use, mz‐visco‐NPS reveals a remarkable single‐cell heterogeneity that will otherwise be masked by ensemble average.

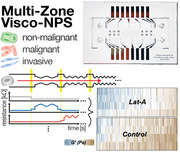

## 1 Introduction

Cells are intrinsically dynamic viscoelastic materials, consisting of elastic biopolymers and viscous fluids that comprise the cytoskeleton and the cytoplasm, respectively.^[^ ^1^, ^2^, ^3^ ^]^ Fluctuations in a cell's viscoelastic phenotype have been correlated with a range of biological processes—from cell growth, motility, and behavior^[^ ^4^, ^5^, ^6^ ^]^ to the epithelial‐to‐mesenchymal transition.^[^ ^7^ ^]^ Previous studies described how viscoelasticity can reflect the metastatic potential in breast epithelial cells,^[^ ^8^ ^]^ assess cell viability after cryopreservation,^[^ ^9^ ^]^ or monitor nuclear reorganization during osteoblast differentiation.^[^ ^10^ ^]^ Overall, viscoelasticity is an inherent physical trait of cells that serves as an important biomarker of cell state and function,^[^ ^11^ ^]^ and its measurement has a wide range of applications—from understanding fundamental biology to potentially diagnosing and monitoring disease.

Despite the fundamental importance of cellular viscoelasticity, a major challenge to its broad adoption at the bench‐side or in the clinic is the accessibility of measurement platforms. The gold standard, atomic force microscopy (AFM),^[^ ^12^, ^13^ ^]^ can provide extensive information on the viscoelastic properties—the elastic modulus (G′) and viscous modulus (G″)—of a single cell at different frequencies; however, throughput is extraordinarily low at approximately ten cells per hour. Furthermore, AFM viscoelastic measurements can be heavily influenced by the substrate to which a cell is adhered.^[^ ^7^, ^14^ ^]^ While they are capable of measuring suspended cells, optical stretchers or tweezers^[^ ^15^, ^16^, ^17^ ^]^ have a throughput of 50 cells per hour,^[^ ^18^, ^19^, ^20^, ^21^ ^]^ which is only slightly greater than that of AFM. In contrast, recently developed microfluidic techniques that utilize constriction channels,^[^ ^22^, ^23^ ^]^ hydrodynamic stretching,^[^ ^24^ ^]^ or shear flow^[^ ^25^ ^]^ to deform cells have much improved throughputs of up to 6 × 10^4^ cells per hour. While these methods provide parameters such as cell transit velocity through a constriction or morphology ratios, e.g. relative deformability, they utilize expensive, complex optical hardware. We, ourselves, previously developed visco‐node‐pore sensing (visco‐NPS),^[^ ^7^, ^26^, ^27^, ^28^, ^29^, ^30^, ^31^ ^]^ a moderate‐throughput (up to 600 cells per hour) microfluidic platform for cellular viscoelastic phenotyping. This platform—which is all electronic—measured cells at single frequencies, and the accompanying rheological readouts were effectively a bulk measurement—the frequency‐dependent elastic G′ and viscous G″ moduli could only be determined for populations of cells and not individual cells.

Here, we introduce multi‐zone visco‐NPS (mz‐visco‐NPS), a true single‐cell approach for viscoelastic phenotyping. mz‐visco‐NPS consists of multiple sinusoidal contraction “zones”—each with a unique periodicity—aligned in series. mz‐visco‐NPS measures the viscoelastic response, i.e., elastic G′ and viscous G″, of each cell over a range of frequencies, while still achieving a moderate‐throughput rate of up to 600 cells per hour. We show that mz‐visco‐NPS can detect breast epithelial cells shifting toward a more compliant, less elastic phenotype when subject to actin cytoskeletal perturbation. Additionally, we identify viscoelastic differences among single cells from three different breast epithelial cell lines, MCF‐10A, MCF‐7, and MDA‐MB‐231. We also characterize different zone configurations, exploring how the applied frequency order and strain duration affect our viscoelastic measurements. Finally, we show that mz‐visco‐NPS measurements uncover broad single‐cell heterogeneity and population differences among average‐ and high‐risk primary human mammary epithelial cells (HMECs).

## 2 Results

### 2.1 Microfluidic Design and Working Principle of mz‐visco‐NPS

**Figure 1:**
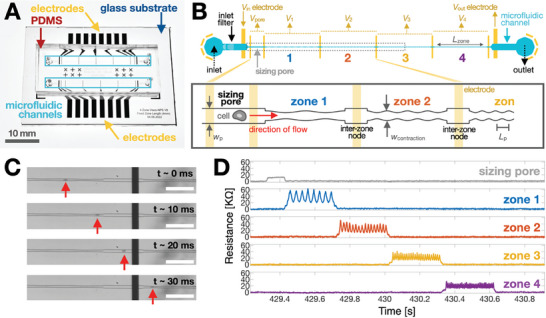
Multi‐zone visco‐NPS overview. A) Photograph of the microfluidic platform, which consists of two independent microfluidic channels (blue boxes) embedded in PDMS and aligned to a glass substrate with prefabricated Pt electrodes and Au contact pads. Scale bar = 10 mm. B) Schematic of a single mz‐visco‐NPS device (blue), consisting of an inlet filter, a sizing pore, and a series of contraction zones (four are shown here). The electrodes (gold) are aligned to the microfluidic channel. The outer electrodes, *V*_in_ and *V*_out_, apply a DC voltage across the channel. Pairs of inner electrodes, labeled as *V*_pore_, *V*_1_, *V*_2_, etc., measure the differential voltage across the sizing pore and each zone. The inset illustrates the sinusoidal geometry of the contraction zones: the width of each zone is described by *w*_contraction_ *= w_o_ + acos(ωt)*, where *w*_o_, a, and *ω* are the initial width, the strain amplitude, and deformation frequency, respectively. Each zone has a constant total length (*L_zone_*) and a different period length (*L*_p_). C) From top to bottom, time snapshots of a single MCF‐7 cell (indicated by a red arrow) exiting one sinusoidal contraction zone (*L_zone_* = 2 mm*, L*_p_ = 300 µm, *w*_contraction_ = 11.25 + 2.75cos(*ωt*) µm), transiting though an inter‐zone node of width *w*_p_ = 21 µm, and finally entering a new sinusoidal contraction zone of different frequency (*L*_p_ = 200 µm). The vertical black bar shown is an electrode. Scale bar = 200 µm. D) Representative resistive pulses caused by an MCF‐7 cell transiting an mz‐visco‐NPS microfluidic channel (*L_zone_* = 4 mm, *L_p_* = 500, 250, 167, 125 µm, *w_contraction_* = 11.25 + 2.75cos(*ωt*) µm) under an applied pressure of 13.8 kPa. The signals are aligned in time; resistive pulses cascade (starting from the sizing pore to zone 1, zone 2, etc.) as the cell transits the microfluidic zones. The periodicity of the signal reflects the periodicity of the applied strain: as the cell transits from zone 1 to zone 4, *L*_p_ is reduced from 500 to 125 µm. Correspondingly, the applied strain frequency increases from ≈30 to 126 Hz.

The mz‐visco‐NPS platform (**Figure** **1**) consists of three primary features: an inlet filter, a sizing pore, and multiple sinusoidal contraction zones in series, each separated by an “inter‐zone” node. Multiple platinum (Pt) measurement electrodes are aligned to the channel inlet, outlet, and inter‐zone nodes (Figure 1B). This electrode configuration helps maintain adequate signal‐to‐noise ratio (SNR) across each contraction zone. The voltage across each zone is recorded using custom hardware that enables multi‐zone data acquisition in an inexpensive, compact form factor that does not rely on optical hardware (Figure S1A, Supporting Information). The two outer‐most electrodes (*V*_in_ and *V*_out_) drive current through the microfluidic channel, while pairs of inner electrodes measure the potential across the sizing pore (*V*_pore_) and each contraction zone (*V*_1_, *V*_2_, etc.) (Figure 1B; Figure S1B, Supporting Information). Pressure‐driven flow is used to move cells through the microfluidic channel (Figure 1C). The inlet filter removes cellular clusters or debris that might otherwise obstruct the sinusoidal contraction zones and is comprised of an array of pillars spaced *w*_p_ apart. *w*_p_ is designed to be two standard deviations greater than the mean cell diameter (Table S1, Supporting Information) to ensure that transiting cells do not experience an applied strain. The sizing pore measures the free‐cell diameter of a cell, *D*_cell_, via the Coulter principle^[^ ^23^, ^26^, ^31^ ^]^ (Experimental Section). As it transits the pore, a cell partially blocks the flow of current, leading to a temporary increase in the pore's resistance (Figure 1D). The magnitude of the change in resistance is proportional to the volume ratio of the cell to the pore, i.e., ∆*R* ≈ *Vol*_cell_/*Vol*_pore_. The contraction zones measure the cell's viscoelastic response to the specific applied strain and frequency in each zone. The zones are positioned in series, each with a periodically changing width, *w_contraction_ = w_o_ + a*cos*(ωt)*, where *w*_o_, *a*, and *ω* are the initial width, the strain amplitude, and deformation frequency, respectively. *ω* is modulated by controlling each zone's period length, *L*_p_. For the experiments described here, we employed a *w*_contraction_ corresponding to an approximate strain of 𝜀 = 0.4 + 0.15cos*(ωt*). Specific channel geometries used for each cell type are detailed in Tables S1 and S2 (Supporting Information). The measured resistance pulses are processed (Figure S1C, Supporting Information) and fitted to a rheological stress‐strain relationship (Experimental Section) to calculate the frequency‐dependent elastic G′ and viscous G″ moduli.

### 2.2 Measuring the Disruption of the Actin Cytoskeleton

**Figure 2:**
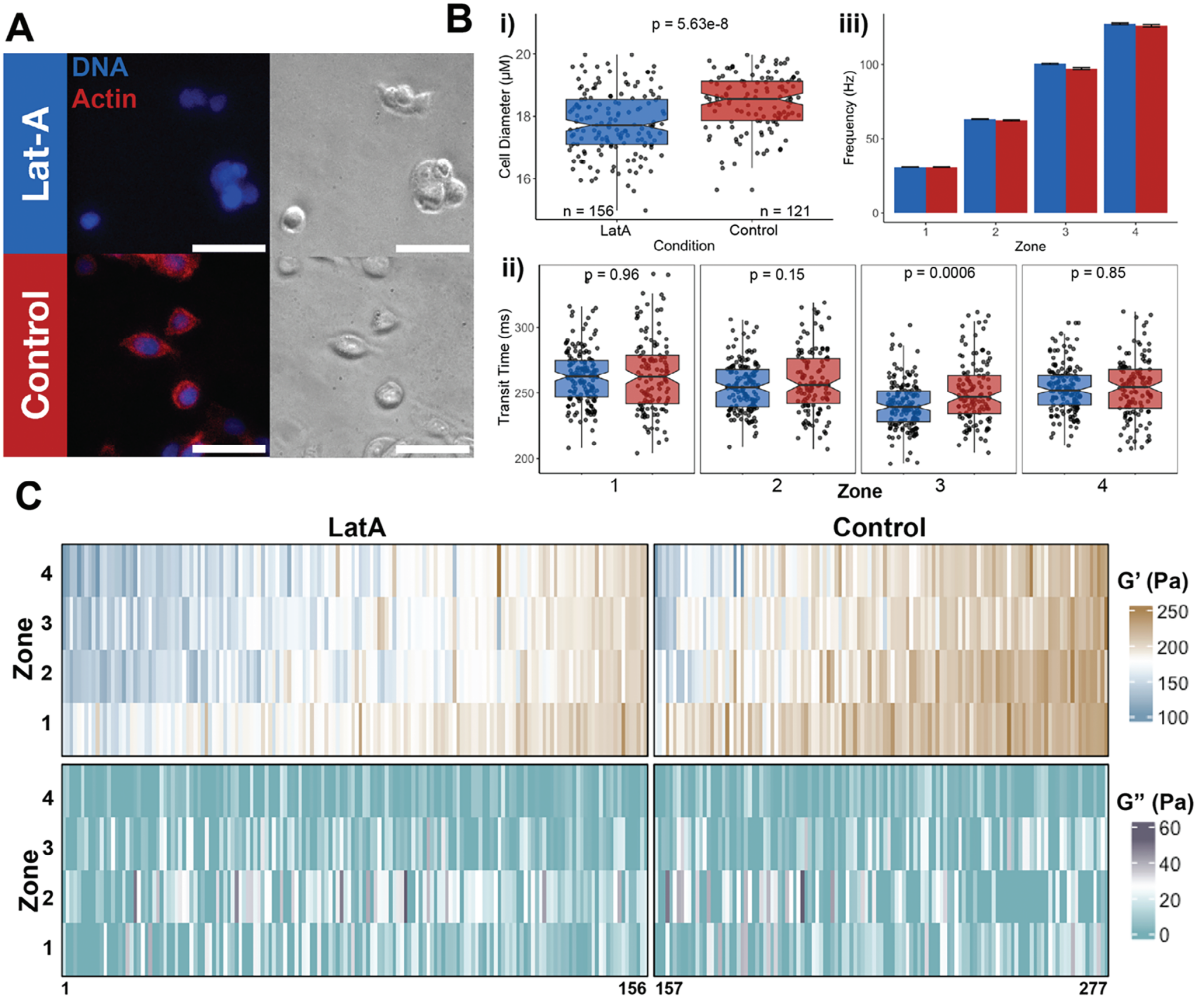
mz‐visco‐NPS detects actin filament perturbations in MCF‐7 cells. A) Fluorescent and brightfield images of Latrunculin‐A (Lat‐A)‐treated (top) and untreated control (bottom) MCF‐7 cells. 4′,6‐diamidino‐2‐phenylindole (DAPI, blue) and rhodamine phalloidin (red) were used to stain the nucleus and actin filaments, respectively. Because Lat‐A disrupts actin filaments, rhodamine phalloidin is not visible in the Lat‐A treated cells. Scale bar = 50 µm. B) Treated (blue) and untreated (red) cells were measured in a four‐zone mz‐visco‐NPS device (shown in Figure 1), with *L_zone_* = 4 mm and *L_p_* = 500, 250, 167, 125 µm. An applied pressure of 13.8 kPa was used. The measured i) cell diameter, ii) cell transit time through each zone, and iii) applied deformation frequency are shown. C) Heatmap of the measured elastic G′ modulus for each single cell in each zone, grouped by Lat‐A‐treatment and untreated control conditions. Each column corresponds to a single cell. D) Heatmap of the measured viscous G″ modulus for each single cell in each zone, grouped by Lat‐A‐treatment and untreated control conditions. Each column corresponds to a single cell. Lat‐A treated cells *n* = 156; Untreated control cells *n* = 121 cells. Error bars in (B, iii) correspond to standard error. p‐values calculated using Wilcoxon rank‐sum test. Notches in the boxplots (B, i and ii) represent the 95% confidence interval of the median.

To demonstrate that mz‐visco‐NPS is capable of distinguishing different viscoelastic properties, we screened MCF‐7 cells whose cytoskeleton was perturbed with the actin polymerization inhibitor, Latrunculin‐A (Lat‐A) (**Figure** **2**A).^[^ ^32^, ^33^ ^]^ We determined that the average cell diameter of Lat‐A‐treated (*n* = 156) and untreated control (*n* = 121) cells populations were 17.8 ± 1.0 µm and 18.4 ± 0.9, respectively (Figure 2B,i). With exception to zone 3, Lat‐A treated and untreated control cells traveled through the platform with similar velocities (Figure 2B,ii). Moreover, the applied frequencies, even in zone 3, were also similar (Figure 2B,iii): a range of 31–127 Hz was achieved across the device, with the lowest frequency in zone 1 and successively higher frequencies in subsequent zones.

In contrast to the untreated control cells, we observed a decrease in elastic G′ in Lat‐A treated cells for all measured frequencies, indicating that they had become more compliant (Figure 2C; Figure S3A, Supporting Information). This is consistent with previous studies that also report a softening of the cell after drug‐induced actin filament disruption.^[^ ^7^, ^34^ ^]^ The smaller diameter and lower elastic G′ modulus of the Lat‐A treated population are all representative of a more compliant cell due to a disrupted actin cytoskeleton. Beyond population‐level differences in elastic G′, we also observed significant cell‐to‐cell heterogeneity, with cells showing a range of elastic G′ from soft to stiff. Nearly 20% of the Lat‐A treated cells had an elastic G′ similar in magnitude to the mean elastic G′ of the control cells. We hypothesize that these particular cells may have recovered more quickly from actin depolymerization. Cells can recover from actin depolymerization in as little as one hour,^[^ ^35^ ^]^ well within the timeframe of our mz‐visco‐NPS experiments (Experimental Section).

When comparing the viscous G″ between the Lat‐A‐treated and control cells (Figure 2D; Figure S3B, Supporting Information), we did not observe significant differences. Broad viscoelastic heterogeneity was observed between the two groups and among single cells. Although approximately 50% of the single cells we measured had a viscosity that fell below our detection threshold value, defined as viscous G″ modulus < 0.1 Pa in at least one zone, we emphasize that mz‐visco‐NPS is still effective in assessing the actin cytoskeleton contribution to a cell's overall elasticity.

When examining the viscoelasticity of Lat‐A‐treated and control cell populations with respect to average strain frequency, we did not observe the widely reported power‐law increase in elastic G′ and viscous G″ with respect to increasing frequencies.^[^ ^1^, ^12^, ^36^ ^]^ One possible explanation is that a power‐law relationship does not always accurately describe cell behavior at low frequencies,^[^ ^37^, ^38^, ^39^ ^]^ such as those achieved in our experiments. Additionally, such power‐law relationships are typically observed over a frequency range that spans multiple orders of magnitude.^[^ ^40^ ^]^ In our experiments, the frequency range spanned less than one order of magnitude. Overall, we consistently observed that cells became less elastic (i.e., showed decreasing elastic G′) with increasing frequency for both Lat‐A and untreated control cells in the limited frequency range we measured (Figure S3A, Supporting Information). To determine whether this behavior is the result of our unique device implementation or design, we assessed the effect of multiple microfluidic geometries on single‐cell viscoelasticity.

### 2.3 Evaluating the Effect of Zone Arrangements on mz‐visco‐NPS Measurements

Because cells are nonlinear materials,^[^ ^41^ ^]^ the frequency order in which they are perturbed can affect their viscoelastic measurement. Thus, we fabricated multiple devices with different zone arrangements and measured MCF‐7 cells to determine the effect of perturbation order and measurement duration.

**Figure 3:**
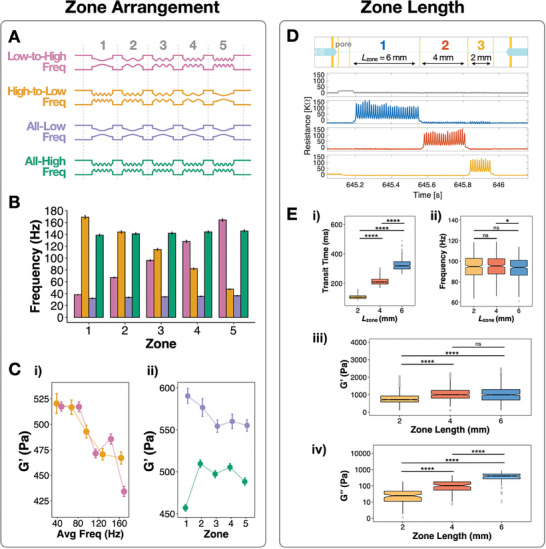
Effect of Channel Geometry on Measured Storage Modulus. A) Four distinct channel geometries were used to test different frequency arrangements. A “Low‐to‐High” frequency device (pink) introduced cells to a low frequency in zone one and then to increasing frequencies in the subsequent zones (from *L*_p_ = 500 to 125 µm). In contrast, a “High‐to‐Low” frequency device (orange) introduced cells to a high frequency in zone one and then to decreasing frequencies in the subsequent zones (from *L*_p_ = 125 to 500 µm). A third “All‐Low” design applied a constant low frequency across all zones (purple) (*L*_p_ = 500 µm), while a fourth “All‐High” design applied a high frequency across all zones (green) (*L*_p_ = 125 µm). Zones for all device geometries had a constant zone length, *L*_zone_ = 2 mm. B) Measured applied strain frequency on MCF‐7 cells for each of the four channel geometries employed. C) i) Comparison of the elastic G′ modulus between the High‐to‐Low and Low‐to‐High frequency arrangement. Elastic G′ moduli decrease with respect to increasing frequency. *n* = 118 MCF‐7 cells for both the High‐to‐Low and Low‐to‐High frequency device measurements. ii) Comparison of the elastic G′ moduli between All‐High and All‐Low frequency arrangement. *n* = 136 MCF‐7 cells for both device types. Storage moduli is greater at the All‐High frequency, than at the All‐Low frequency. D) Schematic (top) of a three‐zone device used to evaluate the effect of zone length on rheological measurements, and a representative example of the signal pulses (bottom) acquired as a cell transits the device arrangement shown. The three‐zone device consists of zone length (*L*_zone_) of 6, 4, and 2 mm, where each zone has an equivalent period length of *L*_p_ = 200 µm. E) The measured i) transit time, ii) applied frequency, iii) elastic G′ modulus, and iv) viscous G″ modulus, versus zone length of MCF‐7 cells; *n* = 361. Although the viscous G“ modulus continues to increase with zone length, the elastic G‘ modulus reaches a plateau with a 6 mm zone length. Error bars in (B) and (C) correspond to standard error; error bars for some frequencies in (B) are too small to be visible. ns, not significant, *p ≤ 0.05, **p ≤ 0.01, ***p ≤ 0.001, and ****p ≤ 0.0001, Kruskal‐Wallis rank test (one‐way ANOVA). Notches in the boxplots represent the 95% confidence interval of the median.

To confirm that perturbation order is not an influencing variable in our measurements, we designed devices in which the applied frequency ranged from low‐to‐high, high‐to‐low, or remained constant (all‐low or all‐high) across five zones (**Figure** **3**A,B). When measuring MCF‐7 cells, we observed no significant differences in elastic G′ based on frequency order (Figure 3C,i). Both the low‐to‐high and the high‐to‐low frequency devices showed decreasing elastic G′ with respect to increasing frequency, just as in our previous experiments. Thus, perturbation order is not a significant factor in mz‐visco‐NPS. Measuring cells in an all‐low frequency device produced a higher elastic G′ than those in the all‐high frequency device (Figure 3C,ii), again consistent with our observation that elastic G′ decreases with increasing frequency. Notably, there was minimal zone‐to‐zone variance in both the “all‐low” and “all‐high” frequency devices, suggesting that cells were not experiencing “material fatigue” despite undergoing repeated stress.

We next determined the effect of zone length (*L*_zone_), i.e., the duration cells are perturbed at a specific strain frequency, on elastic G′ and viscous G″. We designed a three‐zone device with different zone lengths—2, 4, 6 mm, arranged in decreasing order (Figure 3D). Zone lengths greater than 6 mm was not possible to test, given that the total “footprint” of the mz‐visco‐NPS channel would have surpassed the physical length of the glass substrate. The transit time of cells in each zone was linearly proportional to the zone length (Figure 3E, i) and the frequency of strain experienced by cells in each zone was nearly identical (Figure 3E, ii). Although viscous G″ continued to increase with respect to zone length (Figure 3E, iv), elastic G′—the parameter to which mz‐visco‐NPS is most sensitive—plateaued with a zone length of 6 mm (Figure 3E, iii). Thus, for the remaining results discussed below, we employed a microfluidic geometry with a 6 mm zone length.

### 2.4 Comparing the Viscoelastic Properties of Multiple Breast Epithelial Cell Types

**Figure 4:**
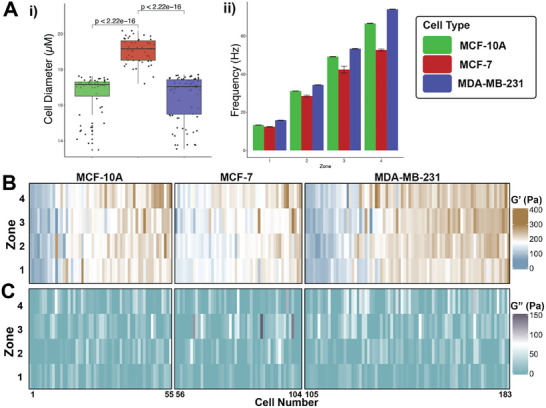
Comparison of Viscoelastic Properties Among Three Breast Epithelial Cell Lines. A) The measured i) cell diameter, and ii) applied strain frequencies for MCF‐10A (green), MCF‐7 (red), and MDA‐MB‐231 (blue) cells in a four‐zone mz‐visco‐NPS device, with *L*_zone_ = 6 mm, and *L*_p_ = 500 to 125 µm. B) Heatmap of the measured elastic G′ modulus for each single cell in each zone, grouped by cell type. Each column corresponds to a single cell. The three cell types have significantly different elastic G′ moduli, with differences most evident in zone 4. C) Heatmap of the measured viscous G″ modulus for each single cell in each zone, grouped by cell type. Each column corresponds to a single cell. MCF‐10A cells *n* = 55, MCF‐7 cells *n* = 48, MDA‐MB‐231 cells *n* = 78. Error bars in A correspond to standard error. *p*‐value calculated using Kruskal–Wallis rank test (one‐way ANOVA).

Using a four‐zone device with a 6‐mm zone length, we measured the viscoelasticity of three breast epithelial cell lines: non‐malignant MCF‐10A, malignant MCF‐7, and invasive MDA‐MB‐231. We measured mean diameters of 16.6 ± 1.1 µm for MCF‐10A, 19.1 ± 0.7 µm for MCF‐7, and 16.5 ± 1.3 µm for MDA‐MB‐231 (**Figure** **4**A,i). All cell lines took approximately 750–1250 ms to transit each zone, resulting in mean applied frequencies of 13, 30, 47, and 65 Hz for zone 1, 2, 3, and 4, respectively, across the three cell lines (Figure 4A,ii).

All three cell lines showed broad heterogeneity (Figure 4B). Malignant MCF‐7s had the lowest average G′ (Figure S4A, Supporting Information). At the highest frequency (zone 4), MDA‐MB‐231′s were more compliant than MCF‐10A's, consistent with their invasive phenotype (Figure S4A, Supporting Information). These results mirror previously published studies.^[^ ^7^, ^12^ ^]^ With regard to viscous G″, we observed similar broad single‐cell heterogeneity with a wide range of phenotypes (Figure 4C; Figure S4B, Supporting Information). Loss tangent (η) is defined as η = G″/G′, where a greater η corresponds to a more viscous phenotype. The loss tangent for malignant MDA‐MB‐231 cells (η = 0.13) was significantly higher than both MCF‐10A (η = 0.053) and MCF‐7 (η = 0.064) cells in the lowest frequency zone (Figure S4C, Supporting Information), indicating that they are more viscous.

Taken together, mz‐visco‐NPS measures distinct single‐cell elastic G′ for MCF‐10A's, MCF‐7′s, and MDA‐MB‐231′s at frequencies from 13 to 65 Hz, and distinct loss tangent (η) at the lowest frequency zone (zone 1).

### 2.5 Uncovering Viscoelastic Differences Among Average‐ and High‐Risk HMECs

**Figure 5:**
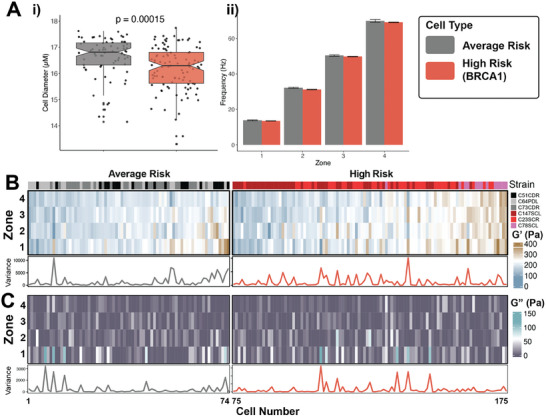
Viscoelastic Properties of Primary Human Mammary Epithelial Cells (HMECs). A) The i) cell diameter, and ii) applied strain frequencies for Average‐Risk (no BRCA1 mutation) HMECs (grey), and High‐Risk (has BRCA1 mutation) HMECs (orange) as measured by a four‐zone mz‐visco NPS device, with *L*_zone_ = 6 mm, and *L*_p_ = 500 to 125 µm. B) Heatmap of the measured elastic G′ modulus for each single cell in each zone, grouped by risk. HMEC strains are as indicated. C) Heatmap of the measured viscous G″ modulus for each single cell in each zone, grouped by risk. HMEC strains are as indicated. The line plots below each heatmap in (B) and (C) show the unit variance of elastic G′ and viscous G″, respectively, across the four zones measured for each single cell. Average Risk HMECs *n* = 74, High Risk HMECs *n* = 101 Kruskal‐Wallis rank test (one‐way ANOVA). Notches in the boxplots represent the 95% confidence interval of the median.

HMECs were screened for differences in viscoelastic phenotypes across six primary strains: three from average‐risk women (aged 33–40 years) without any known breast cancer‐causing germline mutations, and three from high‐risk women (aged 24–35 years) with BRCA1 germline mutations (Table S3, Supporting Information). These HMECs are normal, finite lifespan cells, not cancer cell lines or tumor cells. We employed a four‐zone device with *L*_zone_ = 6 mm. The average‐risk HMECs were larger in diameter (16.6 ± 0.8 µm) compared to the high‐risk HMECs (16.2 ± 0.9 µm) (**Figure** **5**A, i). All HMECs were perturbed at similar frequencies, ranging from 15 to 71 Hz (Figure 5A, ii). We observed broad heterogeneity in the elastic G′ moduli at the single‐cell level in both risk groups (Figure 5B; Figure S6, Supporting Information). At the population level, high‐risk cells were overall more compliant than average‐risk cells (Figure S5A, Supporting Information). Furthermore, an interquartile range analysis of single‐cell data across the zones for both risk groups revealed significant changes in the elastic G′ between high‐risk and average‐risk cells in zone 4, the highest frequency we measured (Figure S5B, Supporting Information). No similar single‐cell and population‐level differences were observed with the viscous G″ measurements (Figure 5C; Figure S6, Supporting Information). To quantify heterogeneity, we examined the unit variance in the elastic G′ and viscous G″ moduli across the four measured frequency zones for each single cell (Figure 5B, C). When comparing high‐risk strains to average‐risk strains, no significant difference in single‐cell viscoelastic heterogeneity was observed between the two risk groups.

## 3 Discussion

We report an all‐electronics‐based, microfluidic platform—multi‐zone visco‐NPS (mz‐visco‐NPS)—that enables true single‐cell viscoelastic measurements. mz‐visco‐NPS uniquely employs multiple sinusoidal contraction zones, each with a periodically changing width and frequency, to measure the frequency‐dependent elastic G′ and viscous G″ response of single cells. Compared to the gold standard AFM,^[^ ^12^, ^13^ ^]^ and newer methods such as hydrodynamic stretching,^[^ ^24^ ^]^ mz‐visco‐NPS has a moderate‐throughput rate of up to 600 cells per hour, obviates the need to consider substrate stiffness by measuring cells in suspension, and does not require high‐speed imaging.

We validated our mz‐visco‐NPS platform by demonstrating that it could measure viscoelastic changes of MCF‐7 cells when their cytoskeleton was disrupted with Lat‐A treatment. Unlike current rheological measurements, mz‐visco‐NPS allowed us to measure the high level of heterogeneity in viscoelasticity among single cells in a population. Intriguingly, we observed that a significant fraction of single cells had an elastic G′ that mirrored the average G′ of untreated cells, possibly indicating that these cells recovered more quickly from actin depolymerization. While further studies are needed to investigate this result, mz‐visco‐NPS is nonetheless capable of uncovering a remarkable level of viscoelastic heterogeneity across single cells that is typically masked by ensemble‐average measurements performed by other rheological platforms.

Using mz‐visco‐NPS, we examined the viscoelastic properties of human mammary epithelial cells that represented the entire continuum of epithelial transformation states: from average‐risk and BRCA1 mutant high‐risk primary epithelial cells (not cancer), to immortal non‐malignant, malignant, and metastatic cell lines. We measured differences in elastic G′ among immortal non‐malignant MCF‐10A, malignant MCF‐7, and metastatic MDA‐MB‐231 cell lines and identified differences in the loss tangent (G″/G′) among all cell lines in the lowest frequency zone, which were consistent with previous viscoelastic measurements of these cell lines at the population level.^[^ ^7^, ^42^, ^43^, ^44^, ^45^ ^]^ Just as in our validation experiments, we identified a high level of heterogeneity within each cell line at the single cell level. When measuring the viscoelasticity measurements of average‐risk and high‐risk HMECs, we again observed significant heterogeneity at the single cell level. Despite this heterogeneity, we could determine that high‐risk HMECs were more compliant than average‐risk cells on the whole. While the high‐risk HMECs we measured bore BRCA1 mutations, we note that Shalabi et al.^[^ ^46^ ^]^ and Miyano et al.^[^ ^47^ ^]^ showed that HMECs with monogenic risk factors such as mutations in BRCA1, BRCA2, or PALB2 all share a common high‐risk phenotype. We thus speculate that so too HMECs with BRCA2 or PALB2 or even HMECS from women who are high risk but have no identifiable germline mutation may also have similar viscoelasticity as the BRCA1^mut^ cells we measured here. The greater physical compliance of BRCA1^mut^ HMECs (as compared to average‐risk HMECs) is congruent with the expected phenotype of a malignant cell. This might suggest that BRCA1^mut^ cells share underlying biomechanical properties consistent with changes that are present in cancer cells. As one example, BRCA1 deficiency has been linked to alterations in biomechanical functions such as cell division.^[^ ^48^ ^]^ Future studies can further explore how viscoelastic differences manifest in high‐risk cells that possess other risk factors, such as family history and age.

We have focused on the remarkable heterogeneity of single cells with regard to their viscoelastic properties and changes to these properties as a result of actin‐filament disruption, whereas mz‐visco‐NPS also could be employed to measure and tease apart the mechanical contributions of 1) different cytoskeletal components beyond actin filaments; 2) nuclear components (e.g., lamins and chromatin); and 3) organelles. With just visco‐NPS (a bulk measurement platform), we previously demonstrated that stabilizing microtubules with paclitaxel increased elasticity of MCF‐7 cells, while destabilizing them with nocodazole decreased elasticity.^[^ ^7^ ^]^ We also quantified, using the same platform, the mechanical transitions of cells, especially changes in the nucleus, as they traverse the cell cycle.^[^ ^7^ ^]^ Given that mz‐visco‐NPS enables *single‐cell* viscoelastic studies at multiple frequencies, performing similar experiments could elucidate the cell cycle‐dependent mechanical changes at the single‐cell level, particularly the dynamic interactions between cytoskeletal, nuclear, and organelle structures. More fascinating, mz‐visco‐NPS could enable studies that explore the contributions of different intermediate filaments (IFs) to the viscoelastic properties of cells. IFs, such as keratins, vimentin, and lamins, are a diverse group of proteins that form an intracellular network which potentially provides additional mechanical support to cells.^[^ ^49^ ^]^ These filaments help maintain cell integrity and elasticity, especially under large mechanical stress and deformation. While the actin‐rich cortex is the primary determinant of cell stiffness when deformations are small, the IF network becomes increasingly important as deformations become larger.^[^ ^50^ ^]^ The effects of IFs on cell viscosity continue to be evaluated and the outcomes are highly context dependent as these networks are highly interconnected.^[^ ^7^, ^51^ ^]^ IFs are highly dynamic, and their mechanical properties can be altered by, e.g., phosphorylation events.^[^ ^52^ ^]^ Consequently, rapid whole cell measurements, such as those provided by mz‐visco‐NPS, could play a critical role in further elucidating the mechanical contributions of different IFs.

Limitations to mz‐visco‐NPS include the limited frequency range at which single cells could be measured and the duration of the applied strain in the contraction zones. These constraints were determined by the physical length of the glass substrates that we employed. Employing a serpentine channel that wraps around the entire area of the glass substrates could overcome these limitations, enabling access to a greater range of frequencies and increasing the duration of applied strain. This could potentially aid in identifying differential phenotypes in the viscous G″, as higher frequencies better capture viscous behavior.^[^ ^53^ ^]^ Currently, mz‐visco‐NPS measures *whole‐cell* viscoelasticity. In contrast, several groups^[^ ^54^, ^55^, ^56^, ^57^ ^]^ have recently developed platforms that can perform multi‐parameter cytometry, measuring for example, polarizability, deformability, cytoplasm conductivity, etc. Because mz‐visco‐NPS takes an electronics‐based approach and is highly adaptable, we could integrate our platform with additional microfluidics and on‐chip sensors to extract additional cellular intrinsic properties, not just viscoelasticity. Furthermore, while we achieve moderate throughput of up to 600 cells per hour, sample rates can easily be further improved. Because we employ an electronic method to measure the viscoelasticity of single cells, we could readily increase throughput by having multiple devices operating in parallel with advanced signal processing to decouple simultaneous readouts.^[^ ^58^, ^59^ ^]^ Finally, we could employ advanced signal processing such as Barker and Manchester coding—all possible because of NPS's unique ability to encode temporal and spatial information in microfluidic channels directly—to resolve coincidence events in a single channel, as we have previously demonstrated^[^ ^58^, ^59^ ^]^

Overall, mz‐visco‐NPS is a new, electronic‐based approach to single‐cell viscoelastic phenotyping. Cytoskeletal reorganizations,^[^ ^60^ ^]^ disruptions in nuclear morphology,^[^ ^61^ ^]^ and even macromolecular behavior in the cytosol^[^ ^62^ ^]^ are all reflected by a cell's viscoelasticity. Because of its versatility, throughput, and ease by which it can be employed, mz‐visco‐NPS could enable a broader adoption of viscoelasticity as a biomarker of a cell's state and function in health and disease.

## 4 Experimental Section

### Theoretical Stress Model for mz‐visco‐NPS Measurements

The working principle behind single‐cell viscoelastic measurements using an electronics‐based node‐pore‐sensor was previously described by J. Kim, et al.^[^ ^7^ ^]^ Fundamentally, the electric resistance across a microfluidic channel was proportional to a transiting cell's diameter—a larger cell will partially block more current than a smaller cell. Measuring the channel resistance can thus quantitatively assess a cell's dynamic deformation in response to the oscillatory stress applied by the contraction channel walls.

If all external forces surrounding the cell were consider and assume constant velocity, the stress on the cell within each contraction zone in mz‐visco‐NPS, ***σ***, can be modeled as:

(1) $$\sigma \;=\;\frac{2\;\Delta P_{\mathrm{avg}}\;w_{\mathrm{contraction}}}{\mu_{f}\;\pi \;D_{d}}$$

where Δ*P*_avg_ is the average pressure difference across a strained cell of a given diameter *d*, *w*_contraction_ is the oscillating width of the contraction channel, *µ*_f_ is the coefficient of friction between the strained cell and channel wall, and *D*_d_ is the diameter of the deformed cell in contact with the channel wall.

*D*_d_ can be determined by noting that the free cell diameter, *D*_cell_ is defined by:^[^ ^7^, ^31^, ^63^ ^]^

(2) $$\;D_{cell}=\;\frac{\Delta R\;D_{eff}^{2}\;L}{\frac{\Delta R\;0.8\;L}{D_{eff}}+R}$$

where Δ*R* is the change in resistance, *R* is the base resistance, *D*_eff_ is the effective diameter of the channel, and *L* is the length of the channel. *D*_eff_ is determined empirically for each device design by calibrating the free cell diameters measured with the device to those obtained by a commercial cell counter (Millipore Scepter 2.0).^[^ ^63^ ^]^ *D*_d_ is related to *D*_cell_ by conservation of volume:

(3) $$\;D_{d}=\;\sqrt{\frac{2\;{D_{cell}}^{3}}{3\;w_{contraction}}}$$

The stress on the cell can then be determined using Equation (1). Using least squares fitting, the modeled stress was fitted to the following standard rheological relationship:^[^ ^7^ ^]^

(4) $$\sigma \;=\;\sigma_{p}+G^{\prime }\varepsilon_{0}cos\;\left(\omega t\right)\;+G^{\prime \prime }\varepsilon_{0}sin\;\left(\omega t\right)\;$$

where is σ_*p*_ pre‐stress, and ε_0_ is pre‐strain. From Equation (4), it can numerically calculate the frequency dependent storage (***G*** ′) and loss (***G*** ″) moduli.

### Device Fabrication

mz‐visco‐NPS consists of a microfluidic channel embedded into a polydimethylsiloxane (PDMS) mold that was bonded to a glass substrate with pre‐fabricated platinum (Pt) electrodes and gold (Au) contact pads. To create the PDMS mold, standard soft‐lithography was employed.^[^ ^64^ ^]^ Briefly, a negative‐relief master was patterned onto a polished silicon wafer using SU‐8 3025 negative epoxy resist (Kayaku Advanced Materials). Sylgard 184 PDMS (Dow Corning) was mixed at a 10:1 w/w ratio of elastomer to curing agent, degassed, poured onto the master, and subsequently cured on a hotplate at 80 °C for 2 h. A slab of PDMS with the embedded microfluidic channel was excised from the master, and inlet and outlet holes were created using a 1 mm biopsy punch (Miltex).

Pt electrodes and Au contact pads were fabricated using standard photolithography. Once a resist template of the electrodes and contact pads were patterned onto a glass substrate, electron‐gun evaporation was used to deposit a thin film of 75/250/250 Å Ti (titanium)/Pt/Au and a lift‐off was performed to remove the excess metal. Gold etchant (Gold Etchant TFA, Transene) was carefully pipetted onto the portion of electrodes to expose Pt in those areas that would make direct contact with the fluid in the channel.

To create a permanent bond between the prepared PDMS mold and the glass substrate with prefabricated electrodes, both the mold and the substrate were exposed to an oxygen plasma (200 mTorr, 30 W, 2 min, Harrick Plasma). After plasma exposure, 30 µL of a 2:1 mixture of methanol and 18 MΩ deionized (DI) water was placed on the glass substrate to facilitate alignment between the PDMS mold and glass substrate under a microscope. Once aligned, the PDMS mold and glass substrate were mated, sealed, and baked on a hotplate at 50 °C for 2 h.

### Data Acquisition Hardware

To measure simultaneously the resistance of multiple zones, a standard four‐point probe measurement was employed. A constant voltage of 5–7 V was applied across the outermost electrodes, and the resulting DC current, *I*_m_, was measured using a transimpedance amplifier (TI OPA172) set to a gain of 10^7^ V A^−1^. The voltage across each zone was independently measured by an instrumentation amplifier (TI INA818) connected to the electrode pairs across each contraction zone (Figure S1B, Supporting Information). The measured voltages, up to six differential voltages (*V*_pore_, *V*_1_, *V*_2_, etc.), and one transimpedance amplifier output voltage (*V*_TIA_) were sampled with a USB‐2627 multi‐channel data acquisition board (Measurement Computing Corporation) at 10^4^ samples/s. The data was transferred by USB to a PC running a custom MATLAB data acquisition interface, available on GitHub (https://github.com/sohnlab/mz‐visco‐NPS). Because all zones were electrically in series, the current through each zone was equivalent to the measured current, *I*_m_. The resistance of each zone (*R*_pore_, *R*_1_, *R*_2_, etc.) was therefore calculated by dividing the measured zone voltage with the single measured current (*R*_pore_ = *V*_pore_/*I*_m_, *R*_1_ = *V*_1_/*I*_m_, etc.).

### Signal Processing and Analysis

Custom MATLAB code was used to process and analyze the measured resistance signals. Briefly, raw resistance signals (Figure S1C, i, Supporting Information) were processed through a low‐pass filter and a moving median window (Figure S1C, ii, Supporting Information). The signals were then normalized to the baseline resistance (Figure S1C, iii, Supporting Information), and distinct cell transit events were identified by tracking signal deviations from the baseline resistance (Figure S1C, iv, Supporting Information). Relevant signal features were then extracted from each identified cell transit event (Figure S1C, v, Supporting Information) to calculate and model single‐cell viscoelastic properties (Figure S1C, vi, Supporting Information). The command‐line interface code used for processing is available on GitHub (https://github.com/sohnlab/mz‐visco‐NPS).

### Cell Culture and Sample Preparation

MCF‐10A (ATCC CRL‐10317) cells were cultured in DMEM/F12 (Gibco 11320033), supplemented with 5% horse serum, 1% Pen‐Strep, 20 ng mL^−1^ human Epidermal Growth Factor, 0.5 µm mL^−1^ hydrocortisone, 100 ng mL^−1^ Cholera Toxin, and 10 µg mL^−1^ insulin. MCF‐7 (ATCC HTB‐22) cells were cultured in RPMI‐1640 (Gibco A1049101) supplemented with 10% fetal bovine serum (FBS) and 1% Pen‐Strep. MDA‐MB‐231 (ATTC HTB‐26) cells were cultured in 50:50 DMEM:RPMI‐1640 supplemented with 10% FBS and 1% Pen‐Strep. All cell cultures were maintained at 37 °C in 5% CO_2_ and routinely passaged, per published protocols,^[^ ^65^ ^]^ once they reached 80–90% confluence. Cells were dissociated with 0.25% trypsin/EDTA for either 3–5 min (MCF‐7 or MDA‐MB‐231 cells)^[^ ^66^, ^67^ ^]^ or 10–15 min (MCF‐10A cells)^[^ ^68^ ^]^ at 37 °C, washed with respective growth media, centrifuged at 200 RCF for 5 min, and re‐suspended at a concentration of 1–2 × 10^5^ cells mL^−1^ in PBS. Single‐cell suspensions were subsequently filtered with a 20 µm filter to remove cellular clumps and then placed on ice prior to being injected into prepared devices for measurement.

Primary HMECs were cultured using previously published protocols^[^ ^69^, ^70^ ^]^ and measured after the fourth passage. HMECs were grown at 37 °C in M87A medium supplemented with cholera toxin and oxytocin. Table S3 (Supporting Information) identifies the strain, age, and breast cancer risk status. Comprehensive details regarding the development and culture of the HMECs used in this study can be found at the Human Mammary Epithelial Cell Bank Website.^[^ ^71^ ^]^

### Pharmacological Treatment for Actin Destabilization

Lat‐A was used to destabilize actin polymerization in MCF‐7 cells.^[^ ^32^, ^72^ ^]^ Lat‐A (Abcam) was reconstituted in ethyl alcohol and added to MCF‐7 cell culture medium at a concentration of 1 µg mL^−1^. Cells were then incubated at 37 °C for 90 min. The concentration and incubation time of LatA was chosen to have a sufficient effect on the cells while also ensuring their viability and was based on previously published work.^[^ ^34^, ^73^, ^74^ ^]^ Cells were dissociated with 0.25% trypsin/EDTA, rinsed with growth medium, centrifuged at 200 RCF for 5 min, re‐suspended in PBS at a concentration of 1–2 × 10^5^ cells mL^−1^, filtered with a 20 µm filter, and placed on ice before injection into devices for measurement. To confirm actin disruption, cells were fixed with 4% w/v paraformaldehyde in PBS for 15 min and permeabilized with 0.2% v/v Triton‐X (Sigma–Aldrich) in PBS for 5 min. Cell nuclei and actin filaments were counter‐stained with 4′,6‐diamidino‐2‐phenylindole (DAPI, Sigma‐Aldrich) and rhodamine phalloidin (Thermo‐Fisher Scientific), respectively, using the manufacturers protocols. Stained cells were imaged with a Nikon Eclipse TE2000 inverted fluorescent microscope.

### Experimental Measurement

Immediately prior to introducing cells for measurement, completed devices were exposed to an oxygen plasma (200 mTorr, 30 W, Harrick Plasma) for 2 min to render the PDMS channel hydrophilic. After plasma treatment, a nonionic surfactant solution of 2% v/v Tween‐20 in PBS was incubated in the channel for 5 min to prevent potential biofouling.^[^ ^75^ ^]^ Finally, pressure‐driven flow (20.7–27.5 kPa for MCF‐10A and MDA‐MB‐231 cells; 13.8–20.7 kPa for MCF‐7 cells; 20.7 kPa for HMEC; Fluigent MFCS‐EZ) was used to drive cells through the channel.

### Statistics

To determine statistical significance, a non‐parametric Wilcoxon signed‐rank test or Kruskal–Wallis rank test was performed in R. All tests used a significance level of *α* = 0.05. Outlier identification and removal was performed for data points that fall below Q1 – 1.5IQR or above Q3 + 1.5IQR. To determine sample sizes, a power analysis, with means estimated from previous studies,^[^ ^23^ ^]^ was performed to ensure that n ≥ 99 cells provided adequate power (π ≥ 0.80) for all experimental groups.

## Conflict of Interest

Lydia L. Sohn is the awardee of US Patent No. 11,383,241: “Mechano‐node‐pore sensing,” J. Kim, S. Han, and L. L. Sohn, issued July 12, 2022.

## Author Contributions

A.L. and S.H. contributed equally to this work. A. L. and L.L.S. conceived of the project. A.L. developed, fabricated, and optimized the mz‐visco‐NPS platform. A.L. also performed all experiments; A.L. and S.H. analyzed all experiments. A.D., A.L., and M.L. created the multizone hardware. S.H. and M.A.L. conceived, and analyzed the results, of the experiments involving the average‐risk and high‐risk HMECs, which they provided. A.L., S.H., M.A.L. and L.L.S. wrote the manuscript with input from the other authors.

## Supporting information



Supporting Information

## Data Availability

The data that support the findings of this study are available from the corresponding author upon reasonable request.
