# Supplementary material for: Multi‐Zone Visco‐Node‐Pore Sensing: A Microfluidic Platform for Multi‐Frequency Viscoelastic Phenotyping of Single Cells
Source: Adv Sci (Weinh). 2024 Sep 23;11(43):2406013. doi: 10.1002/advs.202406013 (PMC11578343; doi:10.1002/advs.202406013)
Supplement: Supplementary file 1 — Supporting Information [file ADVS-11-2406013-s001.pdf]

ADVANCED SCIENCE
Open Access

Supporting Information

for *Adv. Sci.*, DOI 10.1002/advs.202406013

Multi-Zone Visco-Node-Pore Sensing: A Microfluidic Platform for Multi-Frequency Viscoelastic Phenotyping of Single Cells

*Andre Lai, Stefan Hinz, Alan Dong, Michael Lustig, Mark A. LaBarge and Lydia L. Sohn**

Supporting Information

Multi-Zone Visco-Node-Pore Sensing: A Microfluidic Platform for Multi-Frequency Viscoelastic Phenotyping of Single Cells

Andre Lai†, Stefan Hinz†, Alan Dong, Michael Lustig, Mark A. LaBarge, Lydia L. Sohn*

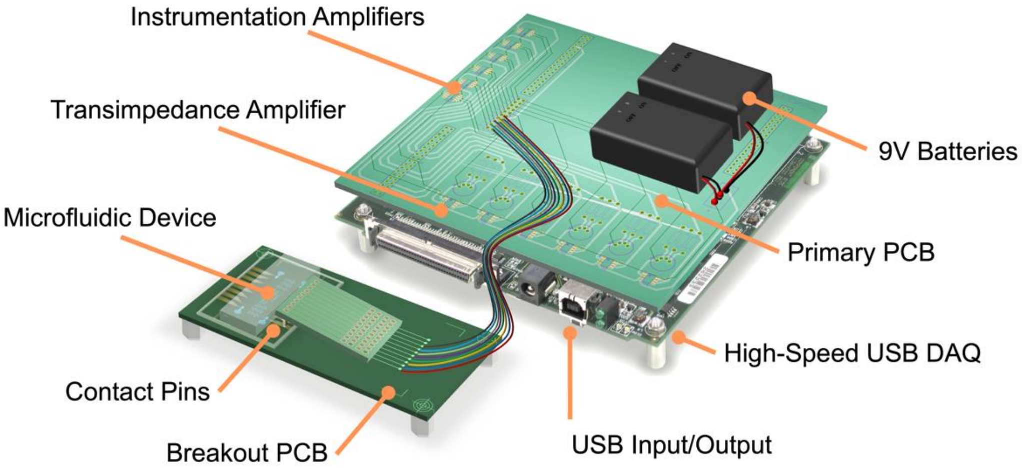

Figure S1A: Schematic of custom data acquisition hardware. The data acquisition hardware consists of a custom battery-powered primary PCB that includes six instrumentation amplifiers for measuring differential voltage and one transimpedance amplifier for measuring current. A separate breakout PCB interfaces with the contact pads on the microfluidic device. The breakout PCB and primary PCB are connected by jumper wires, allowing the circuitry to be reconfigured as needed. This flexible hardware layout ensures measurement compatibility with devices that have different number of zones or a different electrode layout. The primary PCB is connected to a commercial DAQ, which sends power and data though a single USB to a computer.

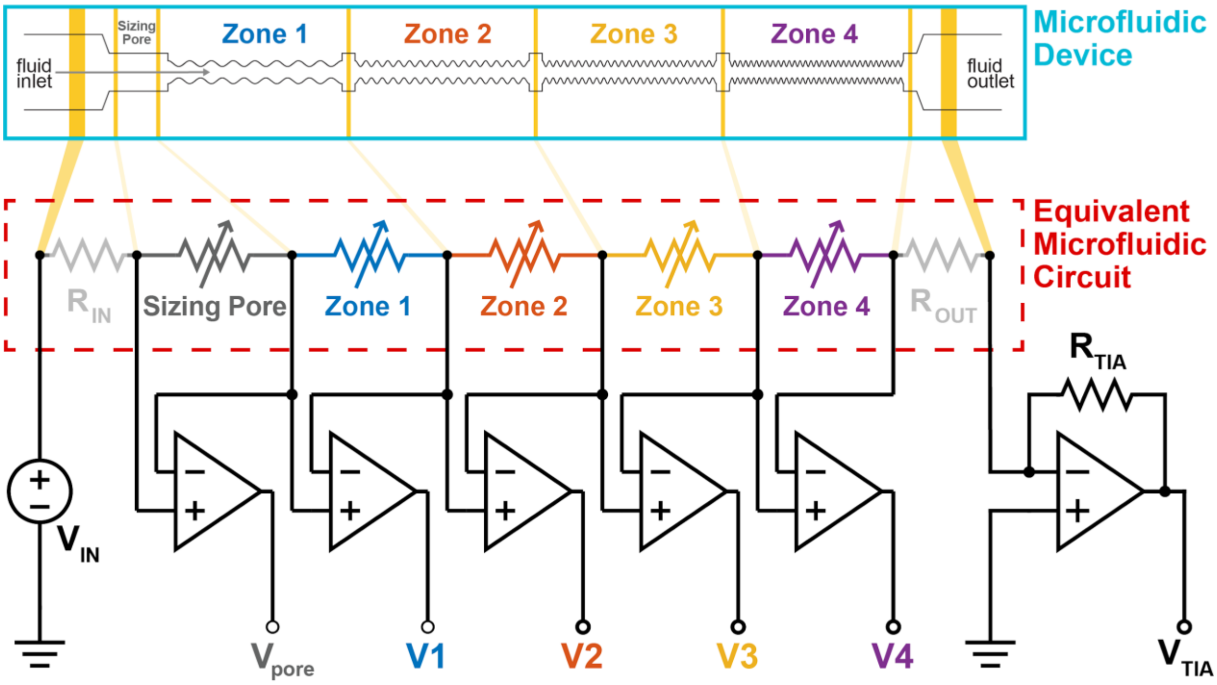

Figure S1B: Electrical Circuit Model for mz-visco-NPS. The microfluidic channel (blue box) can be represented by equivalent circuit components (dotted red line). The channel is biased at the first electrode by V_{IN}. Each zone is modeled as a variable resistor, with fixed resistances at the inlet and outlet, and connected to a differential instrumentation amplifier that measures differential voltage (V_{pore}, V_1, V_2, …) across the variable resistors. A single transimpedance amplifier measures the current across the microfluidic channel at R_{TIA}, with the output V_{TIA}. Because each zone is electrically in series, multiple voltages are measured (V_1, V_2, …), one for each zone, whereas only a single current ($I_m = V_{TIA}/ R_{TIA}$) is measured for the entire device.

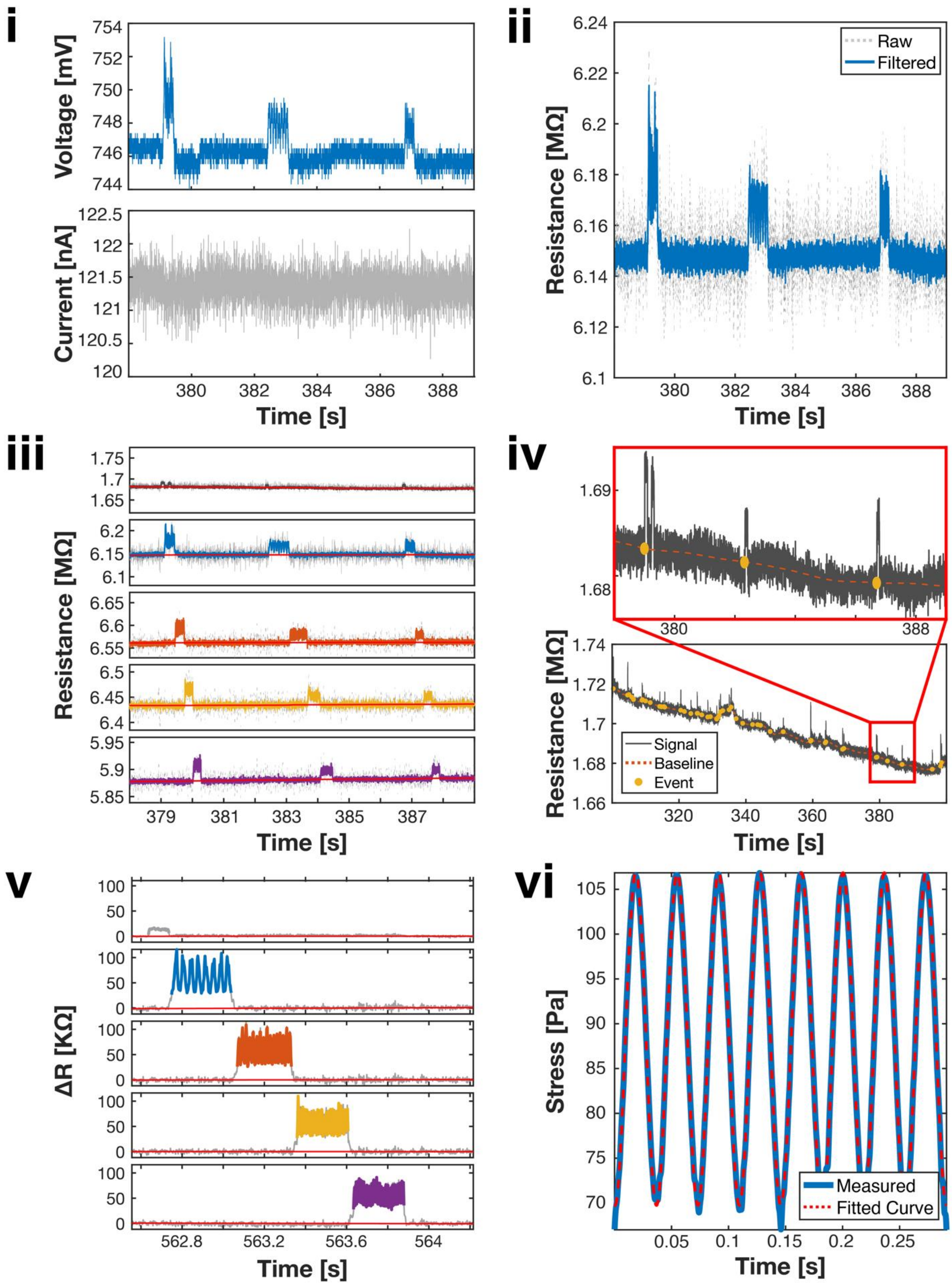

Figure S1C: Signal Processing Pipeline with Custom MATLAB Code. (i) Raw voltage (top) and current (bottom) signals are recorded as cells transit the microfluidic channel. (ii) The resistance across the channel (obtained via Ohm’s law from the raw current and raw voltage) is low-passed filtered to remove high frequency noise. (iii) The baseline resistance (red line) is fitted to the resistance signal for each zone. (iv) By tracking deviations from the baseline resistance, cell transit events are identified and parsed out from the full signal. (v) Relevant

components from each parsed cell transit event are extracted, mainly pulse magnitude and width in the sizing pore, and sinusoidal pulses from the contraction zones. The baseline resistance is shown in (red), the parsed signal in (light grey), and the color highlights (dark grey, blue, orange, etc.) indicate the relevant signal components. (**vi**) The sinusoidal signal component (shown is Zone 1) is used to model the stress on the cell. A standard stress-strain rheological relationship (**Equation 3**) (dotted red line) is then fitted to the modeled stress in order to determine the rheological parameters.

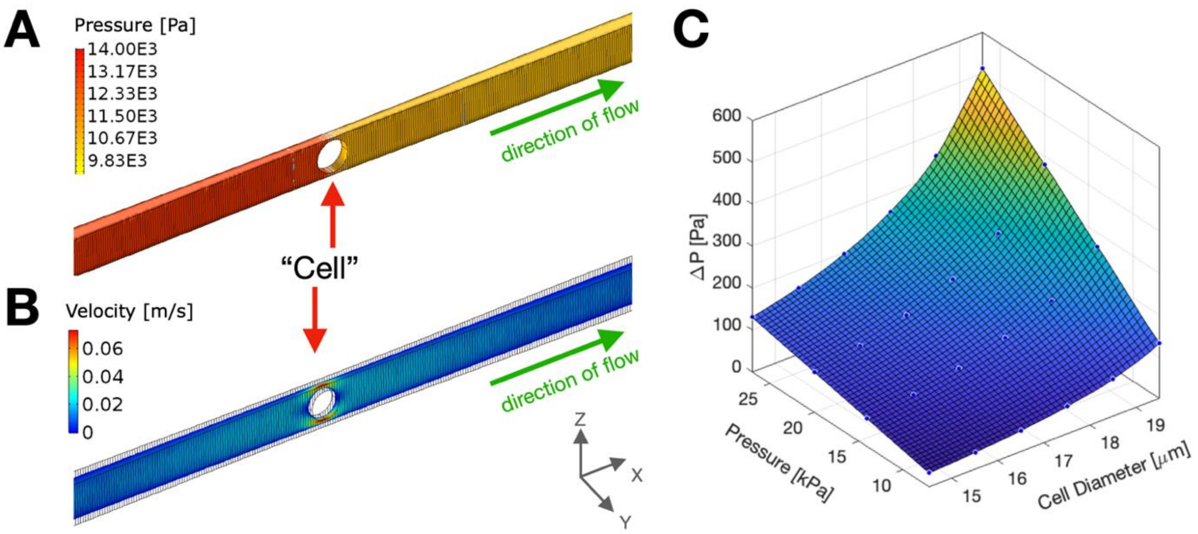

Figure S2: COMSOL Modeling of Cell in a Contraction Zone. . The average pressure across a deformed cell is modeled via COMSOL to estimate the stress on the cell. A three-dimensional, stationary, laminar flow study was performed by importing the exact microfluidic geometry into COMSOL, and appropriately setting an inlet pressure condition and zero-pressure outlet condition. (A) Pressure gradient across the channel with a cell present. The cell is modeled as a cylindrical open gap in the channel geometry. The drop in pressure is measured across either end of the cell (open gap) as the inlet pressure and cell diameter (open gap diameter) parameters are swept. (B) Velocity gradient across the channel with a cell present. (C) Surface plot interpolation of the average pressure across a deformed cell (ΔP_{avg}), which is a function of inlet pressure and cell diameter. See Ref. [7] for further details on ΔP_{avg}.

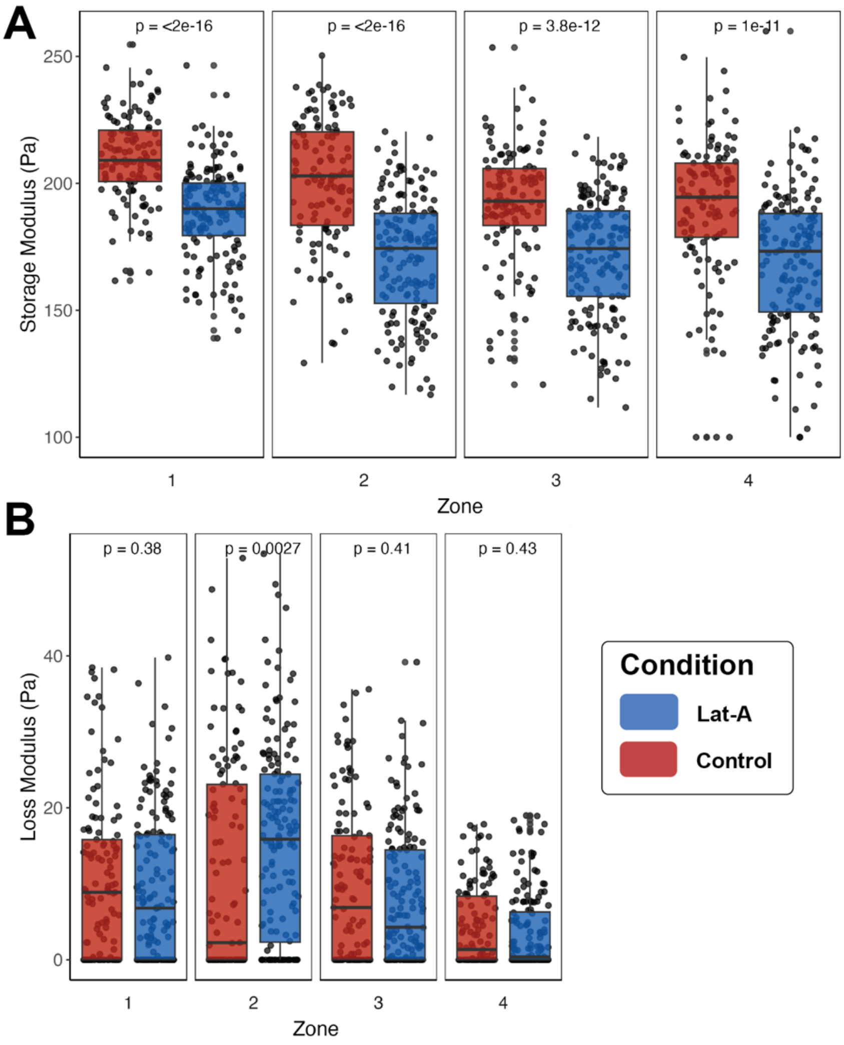

Figure S3: Summary of Average Viscoelastic Properties for MCF-7 Cells Treated with Latrunculin-A (Lat-A). (A) The measured average storage modulus and (B) average loss modulus of untreated control and Lat-A MCF-7 cells measured in each zone with an average applied frequency as detailed in **Figure 2** (**B, iii**). As expected, Lat-A treated cells have a reduced average storage modulus as compared to that of untreated control cells.
Lat-A treated cells *n* = 156; Control cells *n* = 121. p-values calculated using Wilcoxon rank-sum test. Bars correspond to interquartile range.

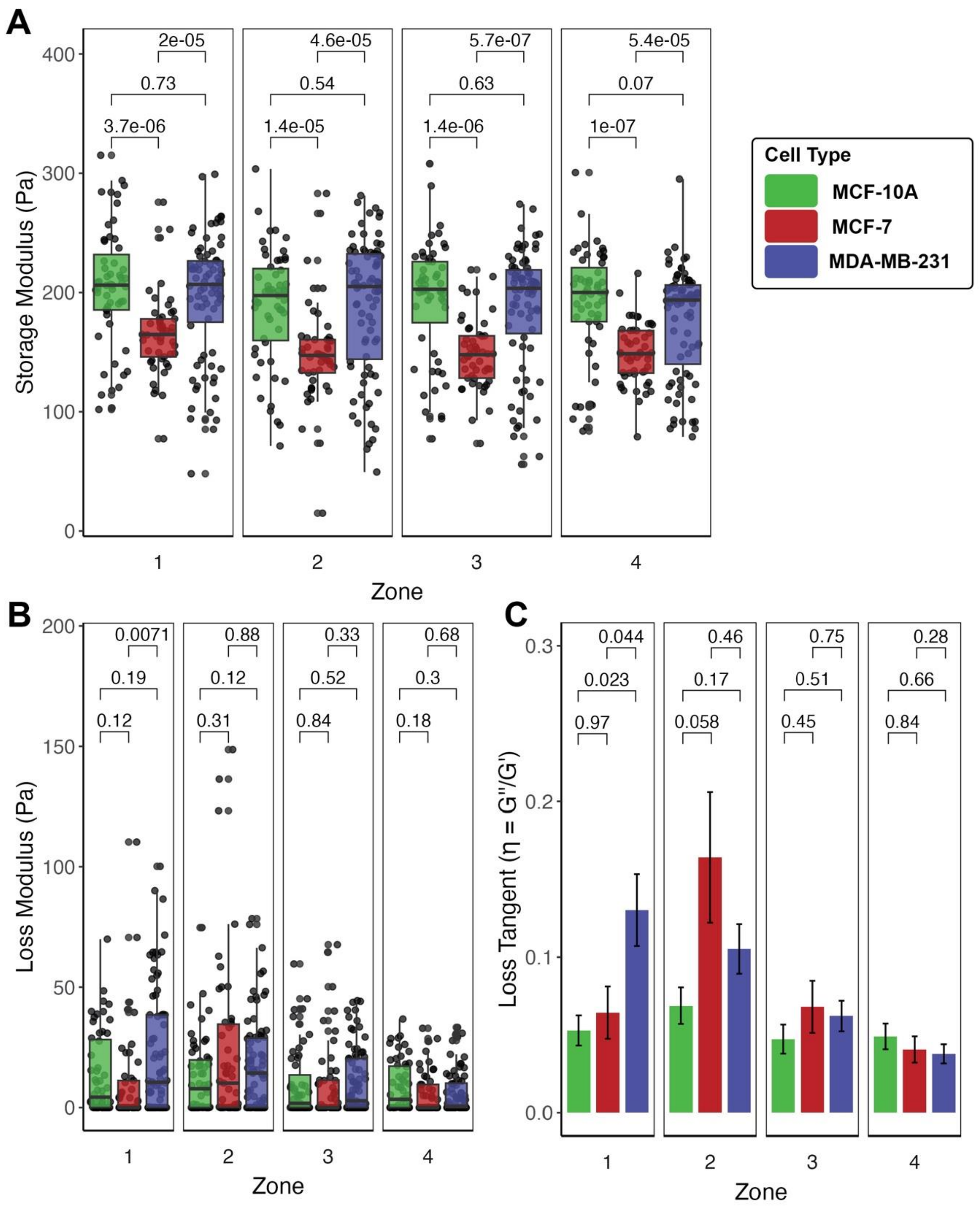

Figure S4: Summary of Average Viscoelastic Properties for 3 Breast Epithelial Cell Lines. (A) The measured average storage modulus (G') and (B) average loss modulus (G") of MCF-10A, MCF-7, and MDA-MB-231 cells, measured in each zone with an average applied frequency detailed in **Figure 4** (**A ii**). MCF-10A cells have a greater average storage modulus than MCF-7 cells, as expected. MDA-MB-231 cells have an average storage modulus between those of MCF-10A and MCF-7 cells, also as expected. (C) The measured loss tangent, defined as η=G"/G', for MCF-10A, MCF-7, and MDA-MB-231 cells, measured in each zone. In the lowest frequency zone (Zone 1, avg freq. = 13 Hz), mz-visco-NPS measures a significantly different loss tangent among all cell lines. MCF-10A cells n = 55, MCF-7 cells n = 48, MDA-MB-231 cells n = 78. p-values calculated using Wilcoxon rank-sum test. Bars correspond to interquartile range.

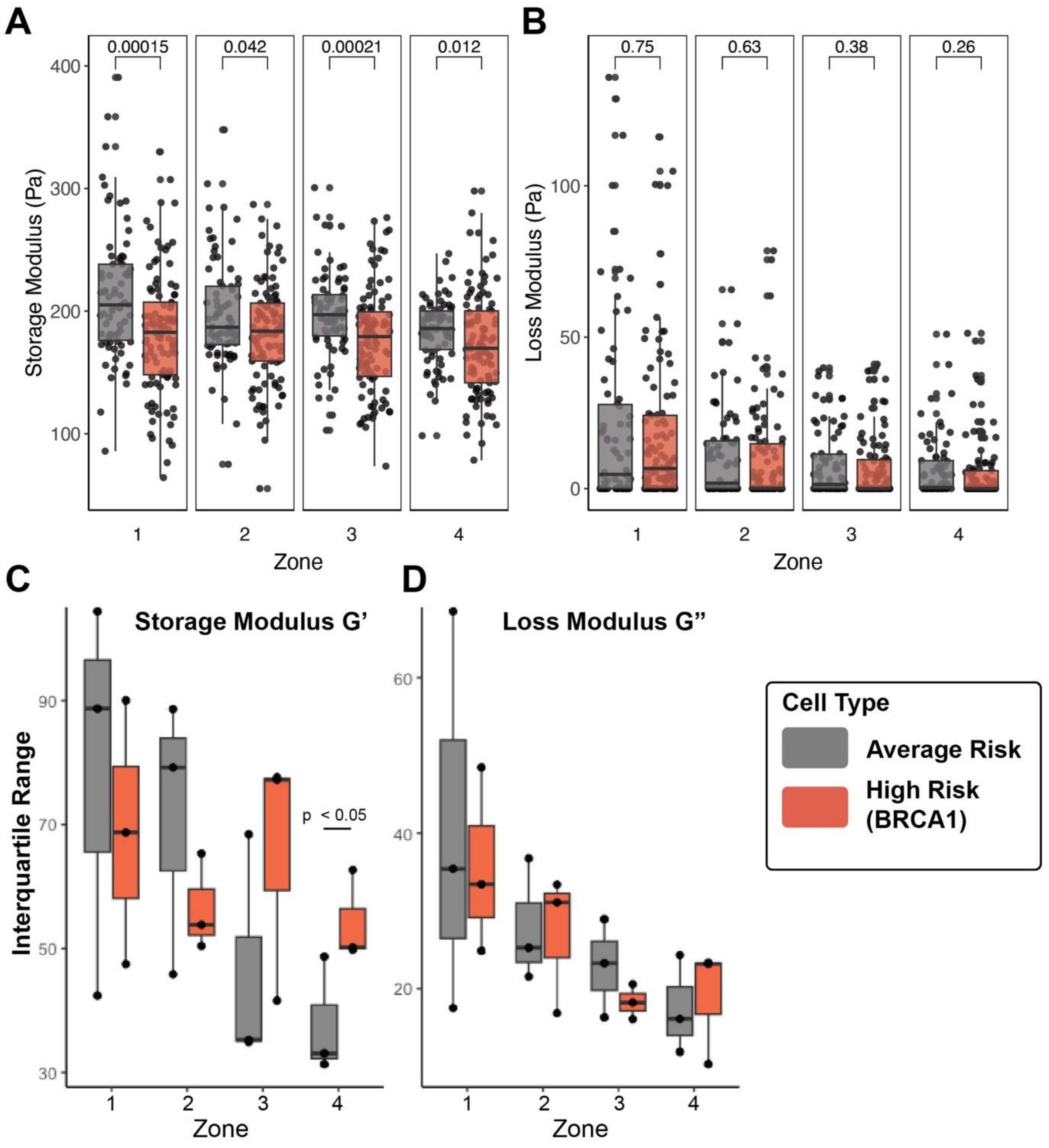

Figure S5: Differences in Viscoelastic Properties for Six Primary Human Mammary Epithelial Cell (HMEC) Strains: average-risk strains (33-40 years old) and high-risk strains (24-35 years old, BRCA1). (A) The measured average storage modulus (G') and (B) average loss modulus (G") of average-risk and high-risk cells, measured in each zone with an average applied frequency detailed in **Figure 5A,ii**. (C) The measured interquartile ranges of average storage modulus (G') and (D) average loss modulus (G") of average-risk and high-risk cells per patient, measured in each zone with an average applied frequency detailed in **Figure 5A,ii**.

Average Risk HMECs n = 74, High Risk HMECs n = 101. Kruskal-Wallis rank test (one-way ANOVA).

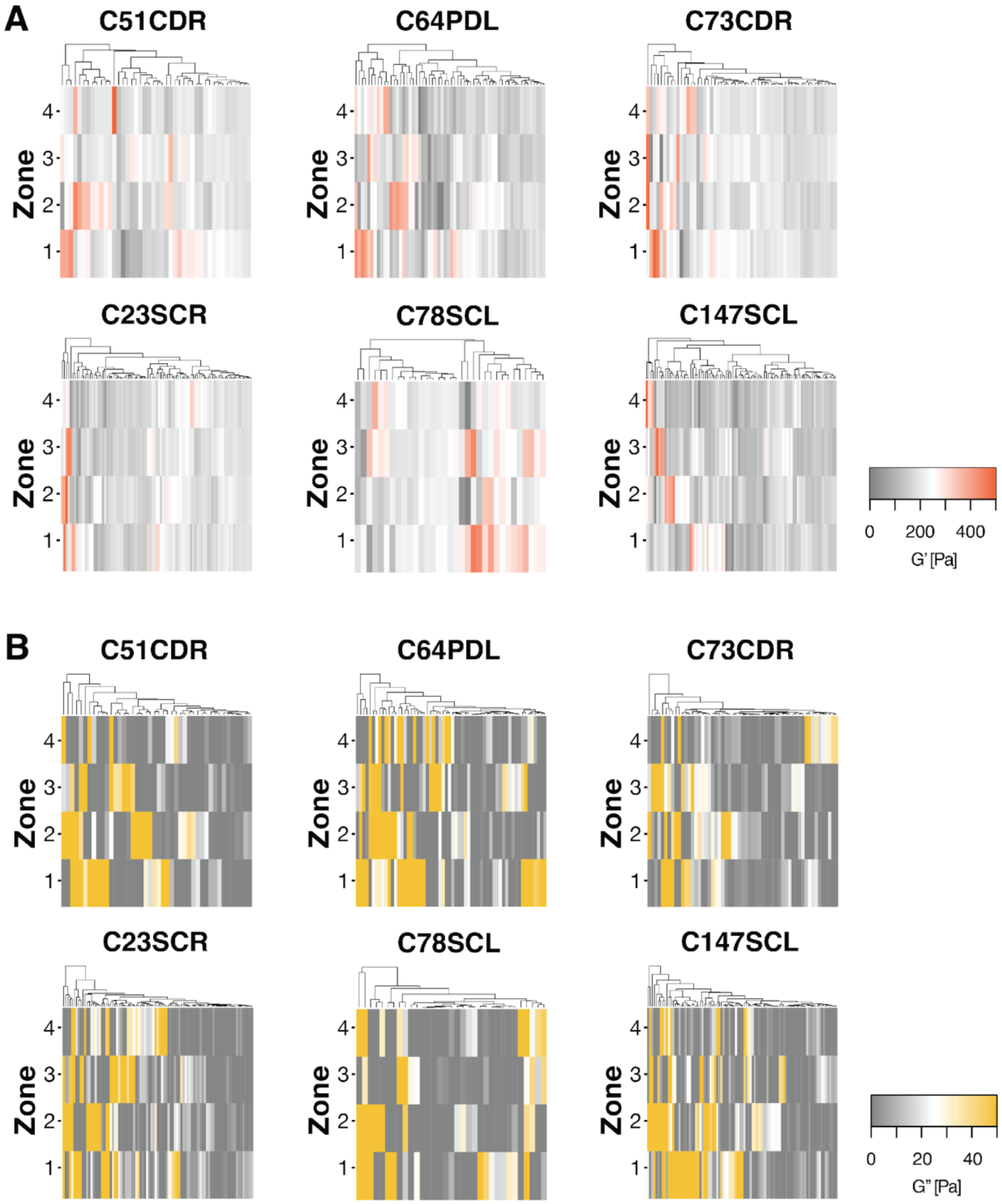

Figure S6: HMEC Viscoelastic Properties Faceted By Strain. (A) G' and (B) G" for Six Primary Human Mammary Epithelial Cell (HMEC) Strains: average-risk strains (33-40 years old) and high-risk strains (24-35 years old, BRCA1). Heatmaps shown with hierarchical clustering; no significant clusters were found. Sample sizes: C51CDR *n* = 44; C64PDL *n* = 62; C73CDR *n* = 57; C23SCR *n* = 89; C78SCL *n* = 33; C147SCL *n* = 91.

SUPPORTING TABLES

Table S1. Microfluidic channel dimensions and strain applied to each cell type. w_p corresponds to the width of the inter-zone nodes and $w_{contraction}$ is the contraction channel width. D_{cell} is the free cell diameter (mean ± standard deviation), and ε is the applied strain to the cells.

<table>
<tr><th>Cell Type</th><th>Avg. D_{cell} [µm]</th><th>W_p [µm]</th><th>$W_{contraction}$ [µm]</th><th>Height [µm]</th><th>ε</th></tr>
<tr><td>MCF7</td><td>19.1 ± 0.69</td><td>21</td><td>11.25 + 2.75$cos\ (wt)$</td><td>32.97 ± 1.91</td><td rowspan="4">0.4 + 0.15$cos\ (wt)$</td></tr>
<tr><td>MDA-MB-231</td><td>16.5 ± 1.26</td><td rowspan="3">17</td><td rowspan="3">9.25 + 2.25$cos\ (wt)$</td><td rowspan="3">28.43 ± 1.15</td></tr>
<tr><td>MCF10A</td><td>16.6 ± 1.12</td></tr>
<tr><td>HMEC</td><td>16.4 ± 0.81</td></tr>
</table>

Table S2. Microfluidic channel lengths and calculated effective diameters for 3-,4-,and 5-zone devices. D_{eff} is the calculated effective diameter of the pore. L_{pore} is the length of the sizing pore, L_{zone} is the length of the contraction zone, and L_{p1}, L_{p2}, L_{p3}, … L_{pn}, where n is the zone number, are the period lengths of zone 1, zone 2, zone 3, … zone n, respectively.

# zones	L_{pore} [mm]	L_{zone} [mm]	L_{p1} [um]	L_{p2} [um]	L_{p3} [um]	L_{p4} [um]	L_{p5} [um]	D_{eff} [µm] for W_p = 21 µm	D_{eff} [µm] for W_p = 17 µm
3	1	6	500	200	125	--	--	18.95	16.70
4	1	4	500	250	167	125	--	18.85	16.70
4	1	6	500	250	167	125	--	19.25	16.65
5	1	2	500	286	200	154	125	20.35	16.4

Table S3. Human Mammary Epithelial Cells (HMEC) used in this study.

Spec ID	Media	Freeze-down #	Age	Risk Status
C51CDR	M87A+CT+X	CB318	33	Average Risk
C64PDL	M87A+CT+X	CC475	40	Average Risk
C73CDR	M87A+CT+X	CB317	33	Average Risk
C23SCR	M87A+CT+X	CC340	35	High Risk (BRCA1)
C78SCL	M87A+CT+X	CA993	24	High Risk (BRCA1)
C147SCL	M87A+CT+X	CC507	33	High Risk (BRCA1)
